# Calling it out? A Q Methodological Study of Sexual Harassment Labelling

**DOI:** 10.1177/10778012231209011

**Published:** 2023-10-25

**Authors:** Lisa Lazard, Rose Capdevila, Jim Turner

**Affiliations:** 1 5488 The Open University, Milton Keynes, UK

**Keywords:** sexual harassment, labelling, Q methodology, feminism, sexism

## Abstract

The public condemnation of high-profile men accused of gendered violence in 2017 resulted in an upsurge of women labelling past experiences as sexual harassment. This study used Q methodology to explore understandings of sexual harassment in the UK. Forty participants sorted 62 statements into quasi-normal grids which were factor analyzed (by person). Eight factors were identified which were titled: Sex Not Sexism, Sexualized Discriminations, Victim Voice, Sameness and Difference, Power/Sex, Repeated Power Abuse, Personal Boundaries, and Masculinity and Heterosexism. Taken together, they signal that feminist efforts to articulate the experience of sexual harassment have gained ground during this period.

## Introduction

The 2017 high-profile cases of sexual assault marked an unparalleled cultural moment of resistance against sexual harassment (Lazard, 2020). Those accused, particularly in the early reporting, were held to account in the popular press. These events created space for countless individuals, particularly women, to voice their own experiences, exemplified by the snowballing of #MeToo and #timesup. The widespread labelling, that is the naming of experiences as sexual harassment, is noteworthy because non-labelling has been a concern in this field. Non-labelling refers to the absence or refusal to use the term sexual harassment (Bongiorno et al., 2020). Labelling, however, is complicated because of the multiple ways in which sexual harassment has been made sense of. Given the visibility over the past half a decade of sexual harassment as a significant social problem, this paper explores how it is predominantly understood in the current cultural context in the UK.

As a backdrop to the current study, this paper will begin by discussing the literature on how sexual harassment has been conceptualized. It then introduces Q methodology and how it was used to explore the labelling and non-labelling of sexual harassment experiences. Eight different narratives and their relationship with existing research are explored to discuss the role of feminism in defining and resisting sexist and unwanted sexualized behavior.

### Conceptualizing Sexual Harassment

Conceptualizations of sexual harassment have been shaped by a number of relevant contexts related to the term's emergence in the mid-1970s. Women's activist groups have been credited with coining the term to problematize women's experiences of unwanted sexual and gendered conduct at work (Mackinnon, 1979). Scholarship around the issue treated the term as a crucial step in making sexual harassment visible as a social problem by providing women with a political vocabulary to problematize and resist such unwanted conduct. Central to early conceptualizations of sexual harassment was how the phenomenon was underpinned by sexism (e.g., Wise & Stanley, 1987; Wright Dzeich & Weiner, 1990). Prior to the term's emergence, these incidences had been routinely trivialized, for example, through the male sex drive discourse (Hollway, 1989), which normalizes this behavior as an inevitable consequence of men's biological urge to have sex (e.g., Tangri et al., 1982; Thompson, 2018). The normalization of sexual harassment has been connected to dominant ideas of heterosexuality in which normative masculinity is expected to be agentic and men the pursuers of sex with women.

Women, on the other hand, have traditionally been expected to be more passive in heterosexual relations and act as sexual gatekeepers (Gavey, 2018). Indeed, the sexual harassment of women has been conceptualized as a key site for establishing heterosexual masculine identities—for instance through the wolf whistling at women by groups of men (Thomas & Kitzinger, 1997). Relatedly, the sexual harassment of men and boys is also seen as a policing mechanism to punish and regulate those not embodying heteronormative masculinities (e.g., Scarduzio et al., 2018). The term was thus seen as playing a key role in challenging the normalization of sexual harassment by reframing such conduct as underpinned by sexism and heterosexism and enabled by the unequal distribution of power.

Given that the term sexual harassment had emerged during women's activism around gendered employment inequality, it is perhaps not surprising that the phenomenon became conceptually linked to workplace environments. Indeed, *quid pro quo* harassment at work, in which sexual activity is made a condition of job security, benefits and reasonable treatment (Mackinnon, 1979), has become a somewhat paradigmatic understanding of sexual harassment.

The idea that sexual harassment is characterized by the misuse of power to procure sexual activity has been a recurrent theme in understandings of sexual harassment in both popular culture and academic study. However, research attempting to define and map the dimensions of sexual harassment as a construct have drawn attention to how manifestations of the phenomenon can include sexualized conduct that is not geared towards sexual activity with any particular person (e.g., display of pornography in professional environments, sexual jokes) or even conduct which is not sexual (e.g., gender-based degradations) (e.g., Dresden et al., 2018; Fitzgerald, 1990; Till, 1980). In the literature, there has been an effort to discourage the conflation between sexual harassment and sexual advance. For example, clarifications of the term have included gender harassment, which refers to sexist behavior which is not explicitly sexual (e.g., Dresden et al., 2018; Schultz, 1998, 2018). The term ‘sexual harassment’ thus brought together a range of instantiations of gendered treatment.

### Women's Non-Labelling of Sexual Harassment

The usefulness of the term sexual harassment as a tool for resistance was brought into doubt by a body of research, which burgeoned in the 1990s, that identified a persistent pattern of refusal by women to use the term to label their experiences. The non-labelling of women's experiences has been a key scholarly concern because it was treated as an indicator of the perpetuation of trivializing discourses which undermine women's experiences (Bongiorno et al., 2020). While the trivialization of sexual harassment remains relevant, debates on this issue were more pronounced in the late 1970s to 1990s where the reasonableness of calling an event sexual harassment was frequently questioned in the popular media. These behaviors were more often classified through a ‘boys will be boys’ discourse while the women making them were accused of being oversensitive to ‘normal’ behavior.

Relevant to whether an incident could be reasonably constituted as sexual harassment was the notion of perpetrator intention. For example, in early UK court cases, intention was used as the basis for establishing a claim as sexual harassment (Edmunds et al., 1998). In everyday discourse, the notion of intention has been similarly used to mitigate the culpability of the perpetrator. For example, Marshall (2017) argues that women use perpetrator intention to distinguish between sexual harassment and more socially acceptable but nevertheless unwanted behavior (Marshall, 2017). Indeed, disclaiming intention has been one key way in which sexual harassment is downplayed. For example, claiming sexual harassment was intended as a joke or as a means to progress an intimate relationship have been identified as common ways to trivialize women's experiences. This has been recently recognized and reflected in legal discourses which have rendered intention irrelevant to prioritize victim/survivor voice (Lazard, 2020). To contextualize this point, in the UK Equality Act, 2010, the legal definition of harassment, including sexual harassment, is unwanted conduct that ‘has the purpose or effect of (i) violating B's dignity, or (ii) creating an intimidating, hostile, degrading, humiliating or offensive environment’. As Middlemiss (2019) notes, in the Equality Act, 2010, there has been a shift in emphasis from the purpose of conduct to its effects, giving the perspective of the victim/survivor precedence over and above the intention of the perpetrator.

Particularly during the 1990s and early 2000s, however, labelling experiences as sexual harassment often resulted in women being trivialized, undermined by claims that the perpetrator's behavior was unintentional, or accused of personal failures (e.g., oversensitivity, misinterpretation, troublemaking) (Bongiorno et al., 2020). However, women's refusal to call experiences sexual harassment was also subject to trouble. More specifically, the literature on non-labelling tended to problematize women who declined to use the label as complicit in their own oppression (see, e.g., Adikaram, 2018; Thomas & Kitzinger, 1997). The problematization of non-labelling by women was challenged by research findings which examined how women talked about their experiences of unwanted gendered and sexual conduct. Based on such data, Lee (2001) concluded that findings suggested that non-labelling is not commensurate with acquiescence. Instead, women problematized unwanted conduct using different descriptors such as ‘sexism’ and ‘working in a sexualized environment’ (p. 35). This, Lee (2001) concluded, pointed to the need for an extension of the political vocabulary around women's victimization to allow women to label their experiences in ways that are meaningful to them. Similarly, Epstein (1997) argued that it may be that gender harassment is not described as sexual harassment because many instances are not obviously sexual. She suggested that the use of other terms such as ‘sexist harassment’ might better describe particular experiences (p. 156). Sexist harassment has been used more recently in research, presumably to flag the relationship between sexism and sexualized and gendered behavior (e.g., Thompson, 2018).

It is important to note that implicit within the non-labelling literature is a polarization between non-labelling as ‘bad’ and labelling as inherently ‘good’. As such, labelling has received less scholarly attention. This was addressed in Lazard's 2009 Q methodological study on everyday understandings of sexual harassment. Nine narratives were identified in which sexual harassment was defined as: (i) sexualized behavior distinct from sexism; (ii) sexualized and sexist exploitation of those with less power; (iii) a transgression of equal opportunities for all; (iv) a violation of personal boundary norms; (v) a transgression of social justice; (vi) a repetitive pattern characterized by requests for sex; (vii) sexual intrusiveness; (viii) intentionally coercive; and (ix) dependent on context. The idea that sexual harassment represented an abuse of power was prioritized in seven of these narratives (1, 2, 3, 5, 6, 7 and 9). While not explicitly framed as power abuse, unequal power dynamics were highlighted when sexual harassment was understood as intentionally coercive (viii) or as a form of manipulation when violating boundary norms (iv). A consideration of the operation of power across these narratives signaled how feminist conceptualizations of sexual harassment had become interwoven in everyday understandings of the phenomenon. It should also be noted that while two of the narratives identified in this study (Factors 1 and 4) (Lazard, 2009) positioned sexism as different to sexual harassment, the remaining narratives made various forms of sexism relevant to the phenomenon. However, the relevance of sexism depended on how sexual harassment was contextualized. For example, when constituted as an issue of equal opportunity (Factor 3), a range of sexist practices constituted harassment. However, when sexual harassment was constituted as a sexualized set of heterosexist practices, exclusionary treatment of lesbian women and gay men constituted harassment while non-sexual sexist practices did not (e.g., Factor 8). This study thus highlights multiple complexities that shape whether issues become understood and labelled as sexual harassment.

The recent upsurge of labelling behavior has renewed concerns in the field around the conflation of sexual harassment with sexual advance. Schultz (2018) argues that this was evident in #MeToo posts which have predominantly labelled sexual acts ‘sexual harassment’ and in the 2017 press coverage which emphasized instances of workplace sexual assault. Schultz (2018) suggests that this conflation may result in the non-labelling of sexisms, non-sexual conduct and when harassment takes place outside of work. Schultz's concern is rooted in how sexual harassment, when understood as an expression of sexual desire, has historically worked to uncouple the phenomenon from gender politics. However, the sexualizing of sexual harassment is linked to #MeToo which is itself embedded in discourses of gender equality (e.g., Xiong et al., 2019). Taken together, Schultz's argument points to new complexities shaping understandings of sexual harassment in contemporary times.

### The Present Study

While a substantial body of work has examined the phenomenon of non-labelling sexual harassment, there has not been a consistent focus on the multiplicity of cultural understandings of sexual harassment or on how that focus could extend discussion beyond polarization of labelling and non-labelling practices. Such an examination is timely given that sexual harassment is currently high on the social agenda which has, as mentioned earlier, contributed to an unprecedented pattern of widespread labelling. While this could indicate that non-labelling is perhaps less prevalent than in previous decades, the pattern may also be indicative of an increase of labelling of some forms of harassment rather than others. The present study aims to extend existing scholarship by empirically exploring current everyday understandings of sexual harassment and the implications this may have for challenging it.

## Method

Q methodology is a mixed method approach which has been widely used to study subjective contestable issues (Kitzinger, 1999; Lazard & Capdevila, 2021; Lazard et al., 2011). For example, it has been used to tap into cultural patterns of understanding on topics such as partnership love (Watts & Stenner, 2005), sexual satisfaction (McClelland, 2014), gender conformity and non-conformity (Brownlie, 2006) and domestic violence (Dell & Korotana, 2000). In a Q study, participants are asked to sort a set of statements from most to least relevant to their perspective on the topic of investigation using a quasi-normal distribution grid. Each of the statements is positioned in relation to each other so values are not ‘absolute’ but rather relative. It is not impossible that a specific participant will find themselves agreeing or disagreeing with the vast majority of the statements, but what is important for the analysis is that those that are more representative remain to the right of those that are less representative.

The participant sorts are then factor analyzed and rotated to identify those sorts that have similar distributions. A weighted average of those sorts that correlate highly with each factor is then used to exemplify that factor (for a detailed description of this process see Stenner & Capdevila, 2019; Watts & Stenner, 2012). Each exemplifying Q sort is then interpreted qualitatively. What is important for the analysis of each factor is not just the absolute position of a statement but also the relative position of all the statements in the sort—the distribution as a whole—as mentioned above. This *gestalt* reading allows us to conceptualize each factor as a narrative or understanding of the topic, in this case, sexual harassment.

Of relevance to this study is that Q, by design, does not presuppose polarized understandings of issues. It assumes there are many ways of making sense of a phenomenon that are made up of different elements and represent different subjective understandings. Thus, Q is designed to make all shared understandings or narratives in a data set visible (Roper et al., 2015). For this reason, it has the capacity to identify multiple narratives some of which will be dominant and others of which will be marginal. Q recognizes that the dominance or marginality of a perspective is not directly related to how well represented they are but rather in who can take up the subject positions being offered. For instance, a manager's understanding of sexual harassment might have more impact on the working environment than that of the receptionist, not because of their knowledge or experience but rather because of the relevant power associated with each of those positions. These two aspects of Q, the rejection of polarized understandings and the recognition of both dominant and marginal perspectives, make it particularly suited to exploring complexity in making sense of sexual harassment.

### The Current Study

#### Q Sort

This Q study consisted of 62 statements (see Table 1) which were taken from Lazard’s (2009) Q study described earlier. The statements were previously tested to establish balance (between positive and negative statements), comprehensiveness (coverage of relevant issues) and clarity (statements are easily understandable). While understandings of sexual harassment have changed in the ensuing decades, we would argue that the ideas that underpin this issue, while differently prioritized, remain consistent across this period (Lazard, 2020). The data was collected using an online survey platform, Qualtrics. Participants were provided with an information sheet about the study and a consent form within the online environment. The study invited participants to answer a few demographic questions, complete the Q study (which required them to sort the 62 statements into a pre-set quasi-normal grid that ranged from +6 to −6), and then to respond to some qualitative questions about their sorting choices. This was followed by a debriefing page that also provided contact information for the researcher for queries or should the participant choose to withdraw their data, and an independent party should they want to raise any issues. The study received ethical approval from the researcher's institution.

**Table 1.** Statement Placement by Factor.

Statement	F1	F2	F3	F4	F5	F6	F7	F8
1. A person is only guilty of sexual harassment if they intended to be offensive	−4	**−1**	*−6*	**−1**	*−1*	**−1**	−2	−3
2. Sexual harassment is a series of incidents	−3	−4	*−5*	−1	*−5*	**6**	3	0
3. Treating pregnant women like invalids can be a form of sexual harassment	*−5*	0	−4	1	−3	**3**	0	0
4. Adults can sexually harass children	**4**	**4**	2	**4**	1	−1	*−3*	3
5. Any comment or behavior that undermines a person because of his or her sexual preference can be a form of sexual harassment	*−5*	2	**3**	1	2	0	1	**3**
6. Obscene phone calls can be a form of sexual harassment	3	2	3	3	*−4*	3	3	**6**
7. Wolf whistling can be a form of sexual harassment	**2**	−1	−2	*−3*	1	−1	−1	−1
8. Being called ‘dear’ can be a form of sexual harassment	−2	*−6*	−2	*−6*	**0**	−3	−5	*−6*
9. Pestering someone for sex can be a form of sexual harassment	3	3	4	**5**	**5**	3	4	*2*
10. Children can sexually harass adults	−1	−4	*−5*	**3**	−4	−2	2	−4
11. Suggesting that a woman sleeps around can be a form of sexual harassment	0	1	**2**	0	**2**	−2	*−5*	1
12. A boss who criticizes an employee's work after they’ve said no to sex can be a form of sexual harassment	1	**5**	**5**	**5**	3	**5**	*−4*	**5**
13. Sexual comments that offend a person can be a form of sexual harassment	3	2	2	*0*	**4**	*0*	*0*	*0*
14. There is a difference between sexism and sexual harassment	**6**	5	0	−3	−2	−3	1	*−5*
15. Invading someone's personal space can be a form of sexual harassment	2	0	−1	2	*−6*	0	**4**	−2
16. Calling a man gay because he will not engage in manly activities can be a form of sexual harassment	−2	−2	1	−1	2	−2	*−3*	**3**
17. Sexual harassment can be about the abuse of power	2	0	3	1	**6**	**6**	*−2*	0
18. Beeping a car horn at someone can be a form of sexual harassment	**0**	−1	**0**	−3	*−5*	*−5*	**0**	−3
19. Complimenting a person's looks can be a form of sexual harassment	**0**	*−5*	−2	−2	−3	−4	−3	−2
20. Suggesting that housework is a woman's job can be a form of sexual harassment	*−4*	−1	−1	1	0	−2	**2**	−1
21. Asking someone personal questions can be a form of sexual harassment	**3**	−3	−4	1	−1	*−6*	−1	−1
22. Having pornographic images in the workplace can be a form of sexual harassment	−1	0	0	*−2*	**5**	**5**	3	−1
23. Calling a woman a lesbian because she will not engage in feminine activities can be a form of sexual harassment	*−3*	1	1	0	**2**	0	−2	1
24. A boss that asks an employee to sleep with him or her in exchange for a promotion is sexual harassment	3	5	5	**6**	5	5	*−2*	5
25. The person on the receiving end should be the one who decides whether sexual harassment has occurred	1	1	**6**	2	1	2	*−5*	−3
26. Any comment or behavior that undermines a person because of his or her sex can be a form of sexual harassment	*−4*	**4**	0	−3	−2	1	3	0
27. Touching a person's genitals can be a form of sexual harassment	**6**	**6**	**6**	4	4	*3*	**6**	**6**
28. Only gay people can sexually harass someone of the same sex	−3	−5	−5	−4	−2	0	*−6*	**2**
29. Sexual harassment can happen anywhere	5	4	*0*	4	**6**	1	1	1
30. Flashing can be a form of sexual harassment	4	1	4	5	3	1	**6**	*−2*
31. Hugging someone can be a form of sexual harassment	0	−3	−2	*−5*	**1**	−4	**1**	−4
32. Touching any part of a person's body can be a form of sexual harassment	0	3	**5**	2	2	4	*−1*	1
33. Sexual comments about men's clothes can be a form of sexual harassment	**1**	0	**1**	*−3*	−2	0	**1**	−1
34. A person putting his or her arm around another person can be a form of sexual harassment	**2**	−2	−1	−1	0	*−4*	0	*−4*
35. Flirting can be mistaken for sexual harassment	0	1	−1	**3**	1	**3**	1	*−2*
36. Being called ‘love’ can be a form of sexual harassment	−2	−4	−3	*−6*	−2	**2**	−1	−2
37. Leering can be a form of sexual harassment	1	0	0	2	3	*−1*	**4**	2
38. Obscene emails can be a form of sexual harassment	**4**	1	3	**4**	3	2	*−3*	2
39. Excluding a person from an activity because of his or her sex can be a form of sexual harassment	*−5*	**3**	0	−2	0	−1	2	2
40. Sexual harassment rarely has anything to do with sexual attraction	−1	−3	−3	*−4*	1	*−4*	2	**4**
41. Sexual comments about women's clothes can be a form of sexual harassment	2	−3	1	**3**	−1	−1	−2	*−5*
42. Staring can be a form of sexual harassment	1	−3	1	−2	−1	**2**	−1	*−4*
43. Stroking someone's back can be a form of sexual harassment	**4**	2	2	*−1*	*−1*	1	3	*−1*
44. Suggesting that child-care is a woman's job can be a form of sexual harassment	*−6*	−1	−1	−4	−1	−5	**2**	0
45. Insulting someone by calling them gay can be a form of sexual harassment	−3	**1**	**1**	0	−3	*−6*	**1**	0
46. Touching someone's bottom can be a form of sexual harassment	**5**	4	4	*2*	3	*2*	**5**	4
47. Suggesting that a man sleeps around can be a form of sexual harassment	−1	−1	2	1	*−4*	−3	**5**	−3
48. Being called ‘darling’ can be a form of sexual harassment	−2	−2	−3	**−1**	−3	−2	**−1**	*−6*
49. Touching someone's knee can be a form of sexual harassment	2	0	3	*−5*	1	1	**4**	1
50. Negative comments about a person's looks can be a form of sexual harassment	−2	*−5*	0	0	−4	−3	0	**4**
51. Telling sexual jokes can be a form of sexual harassment	0	−2	*−3*	0	0	1	−1	**2**
52. Suggesting that women should wear feminine clothes can be a form of sexual harassment	−1	−1	−2	0	**2**	−1	*−4*	−1
53. Repeatedly asking someone out for a date can be a form of sexual harassment	1	0	2	1	*−5*	0	2	**3**
54. Touching someone's hand can be a form of sexual harassment	−1	−2	1	*−5*	0	−3	−2	**3**
55. Any comment or behavior that suggests that a person is immature can be a form of sexual harassment	−3	*−6*	−4	−2	−4	−2	0	**1**
56. Friendliness can be mistaken for sexual harassment	−1	2	−2	*−4*	−3	**4**	0	−3
57. Sexual harassment! Don’t be ridiculous! It's only natural for men to make a pass at women	*−6*	−4	−1	−1	*−6*	**4**	−4	0
58. Touching a pregnant woman's stomach can be a form of sexual harassment	−2	−2	−3	−2	−1	*−5*	**0**	*−5*
59. You can only call an incident sexual harassment when sexual comments or behavior are aimed at a person	1	**2**	−4	2	−2	**2**	*−5*	1
60. In this era of political correctness it is all too easy for innocent remarks to be misunderstood as sexual harassment	0	**3**	*−6*	**3**	0	0	*−6*	−2
61. Touching a woman's breasts can be a form of sexual harassment	5	**6**	4	**6**	4	4	*−4*	5
62. Excluding a person from an activity because of his or her sexual preference can be a form of sexual harassment	*−4*	3	−1	0	0	1	−3	**4**

**bold** = highest ranking, *italics* = lowest ranking.

#### Participants

Participants were recruited through social media (including Facebook, Twitter, Snapchat and Instagram). Posts included a few words of introduction and a link to the study. As per above, all ethical checks and consent were carried out within the online environment once participants followed the link. The aim of participant recruitment was to maximize diversity to facilitate capturing multiple understandings of the issue (Stainton Rogers, 1995). In line with this aim, the only criteria for participation were that respondents be over 18 and fluent in English. Twenty-nine women and 11 men (40 in total) with an age range of 18 to 72 (mean age of 35.9) completed the study (see Table 2 for demographic characteristics). All 40 participants indicated that they had been UK residents. Demographic questions included: What is your age? How would you describe your gender? What country and area do you live in? How would you describe your ethnicity? How would you describe your sexual identity/sexuality? Is there anything else you would like to add about yourself?

**Table 2.** Participant Demographic Characteristics.

Demographic descriptions	*N*
Age	
Mean	35.9
Range	18−72
Gender	
Women	29
Men	11
Sexual identity	
Lesbian/gay/bisexual/queer	7
Heterosexual	29
Unsure	1
Prefer not to say	3
Race/ethnicity	
Indian	1
East Asian	1
Black	2
European	1
White Irish	1
White British	21
White (non-specified)	13

### Statistical Analysis

The statistical analysis was carried out using KenQ, an online software program for Q analysis. Principal component analysis was used with Varimax rotation to identify eight orthogonal factors (see Watts & Stenner, 2005 for rationale for this choice). All eight factors had an eigenvalue greater than 1.0 (the variation of a single Q sort), representing a shared narrative, and were thus selected for analysis (see Stenner & Capdevila, 2019 for further details of this process). Together these eight factors accounted for 71% of the total variance3 (see Table 3 for details).

Exemplifying Q sorts for each factor were then constructed using a weighted average of those sorts which correlated highly on each factor (*r* > 0.6) and weakly on the remaining seven factors (r < 0.4).

The eight factors identified in this study have been titled Factor 1: Sex Not Sexism; Factor 2: Sexualized Discriminations; Factor 3: Victim Voice; Factor 4: Sameness and Difference; Factor 5: Power/Sex; Factor 6: Repeated Power Abuse; Factor 7: Personal Boundaries; and Factor 8: Masculinity and Heterosexism.

## Findings

Each exemplifying Q sort was qualitatively analyzed by interpreting the relative meaning of statements (see Table 1 for a list of each statement's position in each factor). The analysis was supplemented by open-ended comments which were optionally provided by participants. The following interpretation will note, in parenthesis, statement numbers and numeric position (e.g., 1: +6).

**Table 3.** Eigenvalue and Variance Explained per Factor.

Factor	1	2	3	4	5	6	7	8	Total
Eigenvalue	8	4.8	4	4	2.4	2	1.6	1.6	28.4
Variance explained	20%	12%	10%	10%	6%	5%	4%	4%	71%

### Factor 1: Sex Not Sexism

The ‘Sex Not Sexism’ narrative clearly distinguishes between sexism and sexual harassment (14: +6). A range of heterosexisms, including using gay identifications as an insult and suggesting that housework and child-care are women's work, was treated as outside the scope of the term sexual harassment (44: −6; 5: −5; 3: −5; 39: −5; 26: −4; 20: −4; 62: −4). As Participant 31 commented ‘There were a lot of things that I would class as discrimination but not as sexual harassment’. Sexual harassment is presented as something that can happen anywhere and is marked by overstepping personal boundaries in adult or adult-child relationships and causing offence by doing so (29: +5; 4: −4; 21: +3; 13: +3). These acts need not be deliberately offensive (1: −4). Specifically, sexual harassment is defined as unwanted sexualized acts such as pestering or pressurizing someone to consent to sex (9: +3; 24: +3) and the touching of genitals, breasts, bottom (27: +6; 61: +5; 46: +5) or stroking someone's back (43: +4). It also extends to lewd behavior such as indecent exposure and technological harassment such as obscene emails (30: +4; 38: +4). There is no sympathy for the idea that these acts reflect natural male sexual behaviors (57: −6).

### Factor 2: Sexualized Discriminations

Like the ‘Sex Not Sexism’ narrative, the ‘Sexualized Discriminations’ narrative distinguishes between sexism and sexual harassment. As such, gendered comments about child-care and housework are not prioritized in this narrative (14: +5; 20: −1; 44: −1). Sexual harassment can happen anywhere (29: +4) and is characterized by unwanted touching, particularly of breasts and genitals, pressurized sexual activity or punishing sexual refusal—all of which arises out of sexual attraction (40: −3). These behaviors are considered undermining and exclusionary acts that are related to inequalities around gender and sexuality (61: +6; 27: +6; 12: +5; 24: +5; 46: +4; 26: +4; 39: +3; 28: −5; 62: +3). Sexual harassment is not limited to adult relationships; this narrative considers adult-child sexual harassment in its definition. (4: +4). The idea that sexual harassment reflects natural male sexual drive or politically correct oversensitivity is not supported (57: −4; 60: +3). Behaviors that do not obviously involve non-consensual sexualized activities are less likely to be treated as sexual harassment (55: −6; 8: −6; 50: −5; 19: −5; 36: −4).

### Factor 3: Victim Voice

The ‘Victim Voice’ narrative prioritizes victim interpretations of their experience (25: +6) over and above the intention of the perpetrator or set criteria for identifying sexual harassment (1: −6; 2: −5; 59: −4). The idea that political correctness makes it easy for innocent remarks to be mistaken for sexual harassment does not hold sway here (60: −6). This narrative positions perpetrators as adults and engages with the complexities of victimization by acknowledging same-sex harassment (10: −5; 28: −5). It is characterized by pressurized sexual activity and reprisal for refusal (9: +4; 12: +5; 24: +5). Touching someone's genitals, breasts and bottom are highlighted but the point is made that touching any part of a person's body can be sexual harassment (27: +6; 61: +4; 46: +5; 32: +5). These are contrasted with unwanted non-sexualized behaviors including treating pregnant women as incapable or asking personal questions, which were less likely to be labelled sexual harassment (55: −4; 21: −4; 3: −4).

### Factor 4: Sameness and Difference

The ‘Sameness and Difference’ narrative recognizes that gendered power relations shape the meaning of experiences in ways that impact men and women differently. As such, sexual comments about clothing are likely to constitute sexual harassment for women rather than men (41: +3; 33: −3). However, this does not presume gender binary understandings of victimization, as same-sex sexual harassment is recognized (28: −4). This narrative also considers victim-perpetrator dynamics that are not typical of academic or popular understandings of sexual harassment, including child perpetrators (4: +4; 10: +3). Sexual harassment happens anywhere and takes the form of technological harassment, indecent exposure, unwanted touching of sexual body parts (e.g., breasts and genitals), pressurized sexual activity and punishment for sexual refusal (61: +6; 27: +4; 29: +4; 38: +4; 30: +5; 12: +5; 24: +6). Sexual harassment often is about sexual attraction and the sexual overtones of such behavior means that flirting, rather than friendliness, could be mistaken for sexual harassment (35: +3; 56: −4). There is support for the idea that political correctness makes it easy for innocent remarks to be misunderstood as sexual harassment (60: +3). Taken together, behavior that is more ambiguous (e.g., touching someone's hand), less obviously sexual (e.g., hugging) or could be construed as friendly (being called ‘love’ or ‘dear’) is less likely to constitute sexual harassment (36: −6; 8: −6; 31: −5 49; −5; 54: −5).

### Factor 5: Power/Sex

The ‘Power/Sex’ narrative positions sexual harassment as an abuse of power between adults (17: +6; 10: −4). It is unsympathetic to the male sex drive discourse (Hollway, 1989) as mitigating this power relation (57: −6). Like the ‘Sex Not Sexism’ narrative, sexualized acts in any context are prioritized including the touching of breasts and genitals, pressurized sexual activity, reprisal for sexual refusal and obscene phone calls (29: +6; 27: +4; 61: +4; 24: +5; 9: +4; 12: +3; 6: +4). Sexual comments that cause offence constitute sexual harassment. However, such comments, particularly those inferring promiscuity, are more likely to be sexually harassing for women than men (11: +2; 47: −4; 13: +4). Sexual harassment can create a hostile environment through the display of pornography in the office (22: +5). These acts are contrasted with those that are less obviously sexual such as invading someone's personal space or beeping a car horn (15: −6; 18: −5; 55: −4; 50: −4).

### Factor 6: Repeated Power Abuse

The ‘Repeated Power Abuse’ narrative defines sexual harassment as recurrent power abuses (17: +6; 2: +6). Employment structures enable the abuse of subordinates through pressurized sexual activity, punishing sexual refusal and displaying pornography in the office (24: +5; 12: +5; 22: +5). Touching any part of a person's body is sexual harassment but breasts and genitals more so (32: +4; 61: +4; 27: +3). As a form of employment discrimination, particular sexisms, such as treating pregnant women as incapable, is presented as harassing (3: +3). That said, unwanted behaviors that are not overtly sexualized are less likely to seen as sexual harassment (21: −6; 45: −6; 18: −5; 58: −5; 44; −5). This is because sexual harassment is seen as rooted in sexual attraction and in the biologization of men's advances to women (40: −4; 57: +4). This introduces ambiguity where friendliness or flirting can be mistaken for sexual harassment (56: +4; 35: +3), differentiating it from the ‘Power/Sex’ narrative. However, acceptable behavior remains disambiguated from sexual harassment by linking the latter with power abuse (19: −4; 31: −4; 34: −4).

### Factor 7: Personal Boundaries

The ‘Personal Boundaries’ narrative recasts the role of gender and highlights the sexual harassment of both men and women as well as same-sex harassment (61: −4; 11: +5; 47: +5; 28: −6). It moves away from workplace sexual harassment and instead focuses on the phenomenon as a violation of the personal (12: −4; 24: −2; 15: +4). Of particular importance are visual displays of indecency such as flashing or exposure to pornography (30: +6; 22: +3; 59: −5) as well as pestering for sex and associated sexual overtures such as touching someone's knee, bottom and genitals (49: +4; 46: +5; 27: +6). These violations can be undermining regardless of gender. For this narrative, political correctness makes it easy for innocent remarks to be mistaken for sexual harassment and so caution is exercised around relying solely on the victim's perspective (60: +3; 25: −5).

### Factor 8: Masculinity and Heterosexism

The ‘Masculinity and Heterosexism’ narrative downplays the role of sexual attraction in sexual harassment scenarios and instead emphasizes the insidiousness of it. This is illustrated by the idea that negative comments about physical attractiveness can constitute sexual harassment (40: +4; 50: +4). This narrative is also critical of heterosexist practices that reinforce heteronormative masculine ideals. Thus, the use of anti-gay sentiment to undermine or criticize men who reject ‘manly’ activities is challenged as a form of sexual harassment (62: +4; 5: +3; 16: +3). Given this, sexism is clearly relevant to the phenomenon (14: −5). Sexual harassment is also characterized by pressurized dating, *quid pro quo* harassment, criticizing work performance, obscene phone calls and unwanted touching (27: +6; 6: +6; 24: +5; 12: +5; 46: +4; 53: +3). These behaviors are contrasted with actions which lack obvious offensiveness such as being called dear or darling, touching a pregnancy bump and hugging (8:-6; 48: −6; 58: −5; 31: −4; 43: −4; 34:-4; 41: −4). Perpetrator intention is not important to defining sexual harassment but equally some caution is required about relying solely on the victim's perspective (1: −3; 25: −3).

## Discussion

How sexual harassment is understood is inextricably interwoven with the likeliness that individuals will label behavior in this way (Goh et al., 2021). The eight narratives identified point to a number of complexities around how sexual harassment is understood in relation to notions of sexism, power and perpetrator intention which, as discussed earlier, have been important concepts in how sexual harassment becomes constituted. Examination of these complexities, we would argue, is important in terms of the implications they may have for challenging sexual harassment.

All eight narratives positioned sexual acts as a defining feature of the phenomenon. This provides an empirical grounding for Schultz’s (2018) claim that sexual harassment has become too narrowly conflated with unwanted sexual advance and sexualized behavior. As discussed earlier, Schultz argued that the sexualization of sexual harassment distracts from sexist and non-sexual forms of the phenomenon. The ‘Sex not Sexism’ narrative identified in this study supports the idea that current social understandings of sexual harassment do not always extend to non-sexual and gender harassment. While these forms of unwanted conducted were less likely to be called sexual harassment, they were not trivialized. Instead they were problematized through the use of other descriptors such as discrimination. Interestingly, the ‘Sex Not Sexism’ narrative strongly resembles the first factor identified in Lazard’s, 2009 study in which sexualized behavior defined sexual harassment and sexisms were problematized as discrimination. This is strongly resonant with Epstein’s (1997) claim that labelling gender harassment as sexual harassment presents difficulty when conduct is not obviously sexual. The use of terms like discrimination does, however, suggest that non-sexual and sexist displays are understood as manifestations of gender inequality. This supports Lee’s (2001) claim that problematization of behavior can and does occur in the absence of labelling an experience as sexual harassment.

Non-sexual sexisms were generally not treated as sexual harassment across narratives, with the exception of the ‘Repeated Power Abuse’ and ‘Masculinity and Heterosexism’ narratives. The ‘Repeated Power Abuse’ narrative treated undermining practices around pregnancy as sexual harassment because it was understood as employment discrimination. The ‘Masculinity and Heterosexism’ narrative was able to prioritize some non-sexual denigrations because it located sexual harassment as a practice which regulates individuals into heteronormative gender identities. According to this narrative, this regulation is not necessarily sexualized as is illustrated by the inclusion of treating gay identities with derision.

Taken together, these narratives suggest that sexual harassment is not consistently understood to encompass the breadth of non-sexual manifestations of sexism. However, a reluctance to extend the breadth of the term should not be conflated with the idea that the sexualization of sexual harassment decouples it from sexism. For example, six of the narratives identified in this study prioritized ideas around gender inequality to make sense of sexual harassment (e.g., Factors 4–8). This is perhaps not surprising given the increased mainstreaming of feminist ideas over the last few years. Indeed, sexual harassment was treated as synonymous with sexism in four of the narratives (Factors, 4, 5, 7 and 8). These findings can be contrasted with the understandings of sexual harassment identified in the comparable 2009 study mentioned earlier. Six of the nine narratives identified in 2009 positioned sexual harassment as different to sexism (Factors, 1, 3, 4, 6, 8 and 9) while two expressed no difference between sexism and sexual harassment (Factors 2 and 7). Taken together, these patterns provide evidence for an increased acceptance of ideas that link together gender, sexualities and sexual relationships with inequality in understandings of sexual harassment.

The link between sexual harassment and inequalities in these narratives implies consideration of the relationship between sexual harassment and power. As discussed earlier, a recognition of the power dynamics at play in normalized, but nevertheless unwanted, conduct was crucial to reconceptualizing such behavior as sexual harassment. Feminist thinking has pointed to the need to explicitly articulate sexual harassment as a power relation, in order to trouble the normalization of unwanted conduct and place the issue on the social agenda (e.g., Mackinnon, 1979; Wise & Stanley, 1987; Wright Dzeich & Weiner, 1990). Given this, it is surprising that the issue of power was strongly prioritized in only three of the eight narratives—the ‘Power/Sex’ narrative, the ‘Victim Voice’ narrative and the ‘Repeated Power Abuse’ narrative. This contrasts with understandings of sexual harassment identified in the 2009 study, in which seven of the narratives explicitly prioritized sexual harassment as an abuse of power (Factors 1, 2, 3, 5, 6, 7 and 9). The de-prioritization of the role of power in the present study may relate to the increased recognition of sexual harassment as a significant social problem in recent years. The problem of sexual harassment has become, in some respects, less debatable because it is now widely considered socially unacceptable. Given this, the de-prioritization of power may reflect a reduced need to explicitly problematize sexual harassment through recourse to power abuse because it is more firmly subject to social censure. This reading is supported by the general lack of sympathy in these narratives to ideas which normalize or downplay sexual harassment.

The idea that sexual harassment is socially inexcusable comes together with notions of perpetrator intention in these narratives. As argued earlier, intention has been widely treated as a means to distinguish harassing from socially excusable behavior (e.g., Marshall, 2017). Across the narratives, however, the perpetrator's intention to cause offence was consistently treated as irrelevant or unimportant to defining sexual harassment. This is a marked shift from the patterns identified in the 2009 study in which the issue of intention was integral to defining instances as sexual harassment in most of the narratives. A move away from the relevance of intention in everyday definitions of sexual harassment may reflect concerns with the effects of problematic gendered behavior rather than the intent to cause harm (see, e.g., Utt, 2013). This, as mentioned earlier, has been embraced in UK legal discourses which have increasingly positioned the issue of intent as irrelevant in order to prioritize hearing the experiences of those harassed.

Moreover, despite the links made between gender inequality and sexual harassment in these narratives, the exclusion of forms of sexist and non-sexual harassment from these understandings warrants unpacking. This is particularly so because many scholars (e.g., Dresden et al., 2018; Thomas & Kitzinger, 1997) have argued for the value of a wide definition encompassing varied expressions of sexism. Given this, a pertinent question is: what is lost if sexual harassment is more widely understood as a sexualized display? One argument is that cultural understandings such as the ‘Sex Not Sexism’ narrative do not preclude problematization using political vocabularies which also flag inequalities such as discrimination.

That said, it is also the case that the sexualization of sexual harassment, particularly within heteronormativity, even when linked to inequality, may well have implications for what is labelled sexual harassment and what understandings of sexual harassment are socially warranted. The foregrounding of unwanted sexual behavior may de-emphasize the complex and nuanced intersectional dimensions of sexual harassment experiences. As Gill and Orgad (2018) argued, while the #MeToo campaign has been applauded for its engagement with the intersectional character of sexual harassment and violence, particularly around class and race, it has nevertheless promoted a gender binary understanding of the phenomenon as something that men typically do to women. This reflects how sexual harassment is constituted using normative heterosexuality more generally. This, Gill and Orgad (2018) argued, undermines ‘broader coalitions of those facing harassment in the face of masculinist dominance, including cis-women, trans men and women and gender non-conforming subjects and queer subjects of colour’ (p. 1319). Such exclusions may complicate both the process of labelling in specific circumstances as well as place limits on which experiences become heard and garner support. For example, the case of Avital Ronnell, a well-known academic and lesbian, became prominent during the #MeToo revelations for sexual harassment of a gay male student. As Ison (2019) argues, public discussions struggled to make sense of the case because it departs from gender binary understandings of sexual violence and sexual advance, and because of this, the case was more contestable that those that fit into heteronormativity (e.g., McCann, 2018).

## Conclusion

We argue that this research makes an important contribution to our understanding of how sexual harassment is made sense of in contemporary culture in ways which do not presuppose polarizations of non-labelling as bad and labelling as inherently good. Q methodology is well-suited to such an endeavor because it is designed to capture multiple snapshots of cultural understandings of the object of study. In this study, as discussed earlier, the statements used to Q sort or map out narratives of sexual harassment were sampled in 2009. While methodologically this enabled direct comparison with the 2009 study findings and, concomitantly, evidence of shifts in understanding, limitations are evident in this approach. Specifically, statements within the sample capturing heterosexism can be considered limited because since 2009 what were formally known as LGBT communities have now been extended to reflect diversity and complexity of LGBTQIA/LGBTQ+ identities and experiences. There remains a relatively small body of research on sexual harassment of LGBTQIA/LGBTQ+ community members. Future research could address these issues by focusing on the ways in which heterosexism becomes relevant to understandings of sexual harassment that depart from the gender binary of sexual victimization.

Nonetheless, the ways in which sexism, power and perpetrator intention are considered and contextualized across the narratives identified in this study appear to signal that feminist efforts to articulate the problem of sexual harassment has gained ground. Specifically, patterns of understanding across these narratives positions sexual harassment as a non-trivial issue and one that is related to social inequalities. This reflects the achievements of feminism in transforming dominant understandings of sexual harassment from an inconsequential feature of everyday life to a political issue. For activists and practitioners working with those who have had problematic gendered experiences, we would suggest that working flexibly with the boundaries around the constitution of these behaviors might facilitate the taking up of these politically.
